# Resolution by Austin District Medical Society

**Published:** 1894-01

**Authors:** 


THE RESOLUTIONS.

"WHEREAS, The war is over; nearly thirty years have elapsed since the South laid down arms, and her people cheerfully accepting the decision of the sword,—returned to their allegiance to the National government; their sins have been forgiven, and those who bore arms in the conflict have been appointed to offices of honor and trust. Distinguished ex-Confederates fill foreign missions,—sit on the bench of even the United States Supreme Court, and the voices of ex-Confederate leaders are heard in the halls of national legislation; the bloody shirt has ceased to wave, the ghost of Rebellion has long since been laid, and peace reigns throughout the land; sectionalism no longer exists, but we are once more a happy reunited people, by the grace of God enjoying the blessings of an enlightened Republican form of government; therefore be it

"*Resolved*, That it is the sense of the medical profession in this section of the loyal State of Texas, as represented by their delegates to the 25th quarterly meeting of the Austin District Medical Society, held in the Capital City of Austin, December 21st, A. D. 1893, that the ruling of U. S. Commissioner of Pensions Lochren that "no one who was connected with the late Confederate army shall be eligible as Medical Examiner on any U. S. Pension Board," is a species of political proscription long out of date, and unworthy an enlightened *free people*; uncalled for, unwise as a policy, unjust to a large class of worthy people innocent of any political sin or offense whatever, a gratuitous insult to the medical profession,—a reflection on the honesty, integrity and capacity of every ex-Confederate surgeon now living; contrary to, and in conflict with the spirit and pretensions of the Civil Service Reform, and calculated to re-awaken bitter sectional feeling, and antagonize an element whose mission on earth is "peace and good will towards men," and who, even during the heat of the unfortunate strife, now happily long past, were non-combatants, intent only on deeds of mercy to friends and foes alike.

"*Resolved*, That the Austin District Medical Society hereby protest against a discrimination so causeless, unwise and unjust; and that we call on our distinguished President to either redress the wrong or let the world know why we are thus disqualified.

"*Resolved*, That the subject be brought before our State Medical Association at its next annual convention in Austin, in April, for consideration and such action as may, in the opinion of that body of representatives of the entire medical profession of Texas, be wise and expedient.

"*Resolved*, That these resolutions be spread upon the minutes of this Society, and that the Secretary be requested to send a copy thereof to the Honorable Commissioner of Pensions at Washington, and a copy to each of the leading papers of the State with the request to publish the same."

